# A case report of aneurysmal bone cyst of the thoracic spine treated by serial anterior and posterior fusion

**DOI:** 10.1097/MD.0000000000017695

**Published:** 2019-11-01

**Authors:** Jongpil Eun, Youngmin Oh

**Affiliations:** Department of neurosurgery, Research Institute of Clinical Medicine, Biomedical Research Institute, Chonbuk National University Medical School and Hospital, Jeonju, South Korea.

**Keywords:** aneurysmal bone cyst, bone tumor, spinal tumor

## Abstract

**Rationale:**

Aneurysmal bone cyst (ABC) is a benign, reactive, non-neoplastic, proliferative, highly vascular osseous lesion. Because of the rarity of aggressive ABC cases, diagnostic and treatment protocols remain controversial and problematic. Treatment of ABC includes surgery, radiotherapy, selective arterial embolization (SAE), and a combination of these modalities. Successful outcomes have been reported, but the technical requirements and complications of each modality are quite different. We report the clinical, radiological, and therapeutic aspects of ABC of the thoracic spine in an adolescent who was treated by circumferential fusion, and we review the published literature.

**Patient concerns:**

An 18-year-old boy was transferred to our hospital complaining of a 2-month history of neck pain.

**Diagnosis:**

ABC of the thoracic spine

**Interventions:**

Six days after SAE, T1 corpectomy was performed via an anterior approach. We performed the operation using the posterior approach 1 week after the anterior approach. Histopathological examination confirmed the diagnosis of ABC.

**Outcomes:**

No neurologic deterioration occurred during the postoperative period. Follow-up X-rays 2 year postoperative showed good bony fusion and alignment.

**Lessons:**

Primary ABC of the spine is a benign lesion with a potential to be locally aggressive and a high rate of local recurrence. The optimal treatment of thoracic lesions is challenging due to their proximity to the spinal cord and nerve roots, and their frequent association with deformity. Surgical resection/curettage, SAE, and radiotherapy can be used alone or in combination. Complete exposure and resection is crucial to avoid the recurrence. Circumferential fusion and reconstruction of stability are also important for the treatment of thoracic ABC, especially in adolescent patient.

## 1 Introduction

Aneurysmal bone cyst (ABC) is a benign, reactive, non-neoplastic, proliferative, highly vascular osseous lesion. It usually affects children and adolescents, with approximately 80% of patients in their first two decades. The incidence of ABC is 0.14 per 100,000 individuals. It usually arises from long bones, but approximately 12% to 30% of lesions involve the spine.^[1,2]^ The lumbar spine is the most frequent site, followed by the thoracic spine. Only 2% of ABCs occur in the cervical spine.^[3]^

Because of the rarity of aggressive ABC cases, diagnostic and treatment protocols remain controversial and problematic. Treatment of ABC includes surgery, radiotherapy, selective arterial embolization (SAE) and a combination of these modalities. Successful outcomes have been reported, but the technical requirements and complications of each modality are quite different. We report the clinical, radiological, and therapeutic aspects of ABC of the thoracic spine in an adolescent who was treated by circumferential fusion, and we review the published literature. Patient has provided informed consent for publication of the case.

## 2 Case report

**Figure 1:**
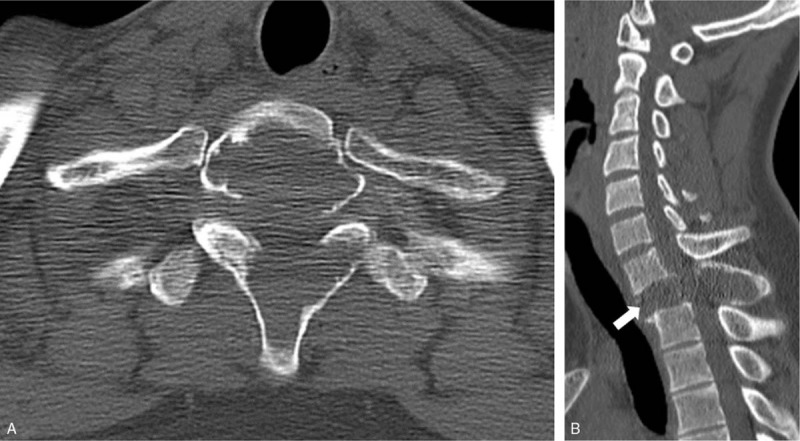
Preoperative computed tomography. (A) and (B) Axial and sagittal image showed osteolytic bony destruction of the T1 body involving the pedicle and spinous process.

**Figure 2:**
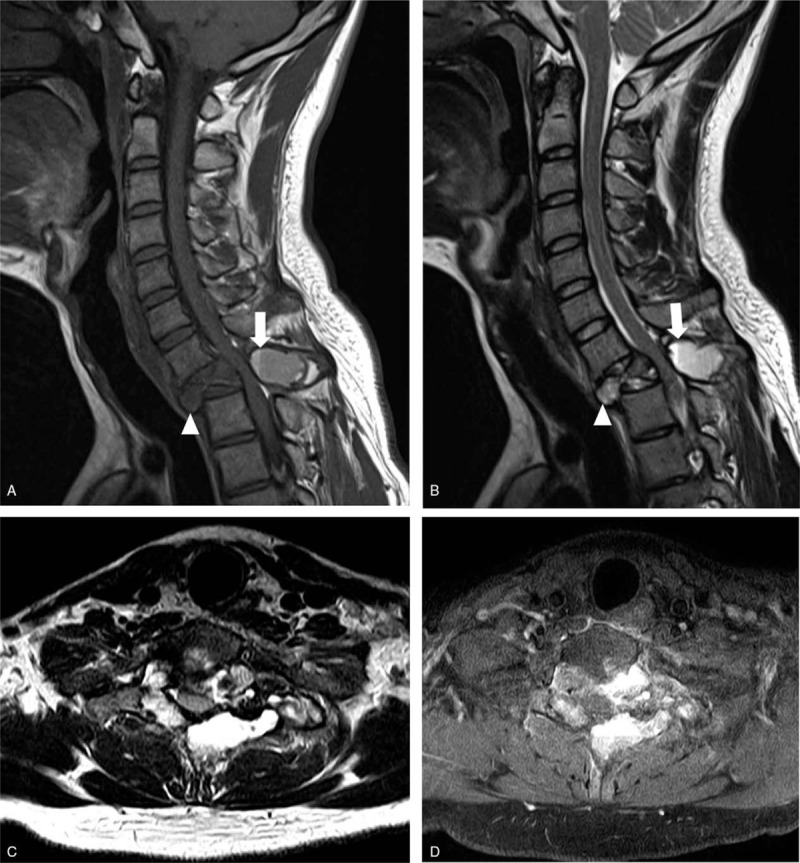
Preoperative magnetic resonance imaging. (A) and (B) T1- and T2-weighted sagittal image reveled a bony cystic mass with internal septation (arrow head) and fluid-fluid levels (arrow) at the T1 vertebra. (C) T2-weighted axial image showed multiple cysts and internal septations, and these cyst walls and internal septations were enhanced on T1-weighted image (D).

An 18-year-old boy was transferred to our hospital complaining of a 2-month history of neck pain. The patient had no history of surgery or trauma and no motor weakness. Computed tomography (CT) of the spine showed osteolytic bony destruction of the T1 body involving the pedicle and spinous process (Fig. 1). Magnetic resonance imaging (MRI) of the spine revealed a bony cystic mass with internal septation and fluid-fluid levels at the T1 vertebra (Fig. 2A and B). The mass was extended to the pedicle and spinous process, and showed heterogeneous signal intensity on T1- and T2-weighted images. The cyst wall and internal septation were enhanced (Fig. 2C and D). There was also a soft tissue mass and cord compression. The T1 vertebral body was destroyed.

**Figure 3:**
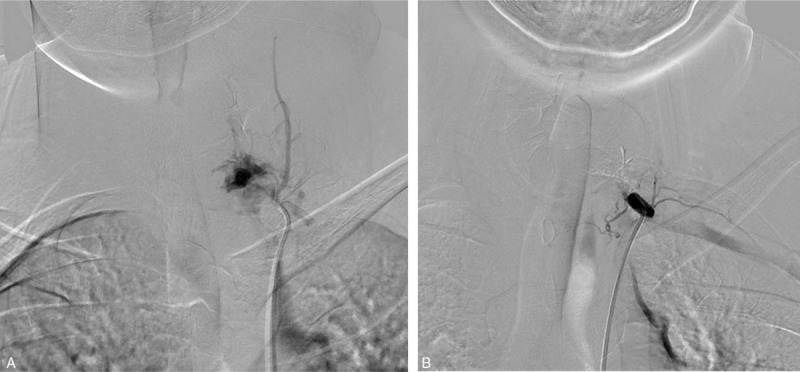
Preoperative angiographic examination. (A) There were several feeders through the thyrocervical trunk. (B) We embolized these feeders with glue.

**Figure 4:**
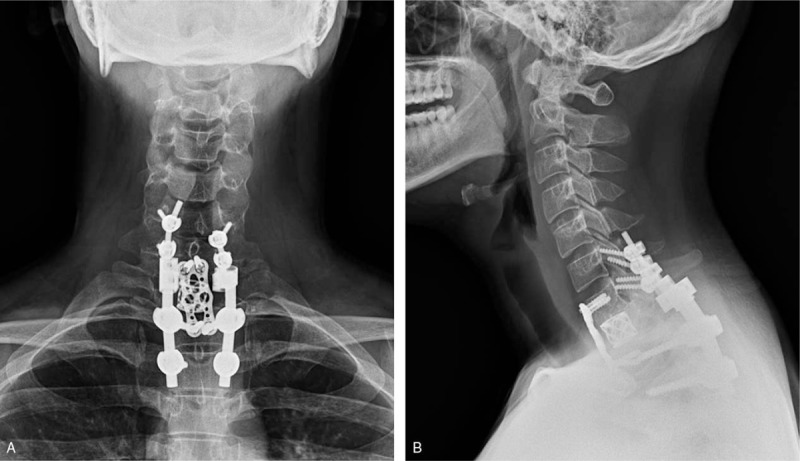
Post-operative X-ray image. (A) and (B) AP and lateral X-ray image showed anterior interbody fusion with titanium graft placement and cervical plating from C7 to T2. And posterior screw fixation was also performed from C6 to T3.

On angiographic examination, there were several feeders through the thyrocervical trunk and we embolized these feeders with glue (Fig. 3A and B). Six days after embolization, T1 corpectomy was performed via an anterior approach. The tumor was pinkish, friable and highly vascular. Although we performed preoperative SAE to prevent significant intraoperative bleeding, massive tumor bleeding occurred during the operation. Bleeding control was difficult because the operative field was narrow, and the tumor could not be fully exposed. After the bleeding controlled, anterior interbody fusion with titanium graft placement and cervical plating from C7 to T2 was performed. We performed the operation using the posterior approach 1 week after the anterior approach. The lesion had involved the T1 pedicle, spinous process and paraspinal muscles. After gross total tumor excision, posterior screw fixation was performed from C6 to T3 (Fig. 4). Histopathological examination confirmed the diagnosis of ABC. His presenting neck pain was improved soon after surgery and no neurologic deterioration occurred during the postoperative period. Follow-up X-rays 2 year postoperative showed good bony fusion and alignment.

## 3 Discussion

ABC is an expansile, tumor-like, osteolytic lesion consisting of a highly vascular honeycomb of blood-filled cavities separated by connective tissue septa, surrounded by a thin cortical bone shell that may expand. It usually affects the pediatric population.^[4]^ Although viewed as a benign lesion, ABC can aggressively destroy and expand bony tissue. Although ABCs most commonly occur in the metaphyseal region of long bones, about 12% to 30% of cases involve the spine.^[1,2]^ The etiology was not fully understood. Previously, Boriani et al^[2]^ suggested that ABC develops from one of the following processes: improper repair of a traumatic subperiosteal hemorrhage; vascular disturbance of the bone; or hemorrhage into a preexisting lesion.

Common presenting symptoms include ill-defined somatic pain, stiffness, and swelling with associated pathologic fracture and bony destruction.^[5,6]^ Most of these cases involve small lesions and only 1 segment of the column.^[7]^ ABC usually arises in the posterior elements of a vertebra, spreads into the pedicles and body, and can expand to the spinal canal, resulting in pathological fractures and neurological deficits. It may occur in bone as a solitary lesion or in association with other tumors, such as giant cell tumors, osteoblastoma, chondroblastoma, chondroid myxoma, fibroma, and fibrous dysplasia.^[8]^

Although diagnosis of ABC can only be confirmed through biopsy, preoperative radiologic evaluation with MRI and CT is useful in diagnosis and surgical planning.^[2]^ The finding of multiple internal septa with fluid-fluid interfaces, which is better observed on MRI, is highly suggestive of ABC.^[9]^ The inside of the tumor contains multiple cysts, commonly with fluid levels and varying signal intensity in T1- and T2-weighted images. These signal differences are caused by different oxidation levels of the blood and blood breakdown products in the cyst fluid.^[10]^

Identification of feeding vessels can be helpful in preoperative embolization, which is required in selective cases to minimize intraoperative blood loss.^[9]^ However, the success of these therapeutic procedures depends on the size of the tumor and the degree of osseous destruction. For larger tumors with numerous supply vessels to embolize, complete devascularization of the tumor is often impossible. Some authors believe that SAE should be considered as the first-line treatment for ABC unless pathological fracture or neurological deterioration is detected or SAE cannot be performed due to technical or safety considerations.^[2,4,11,12]^ In a case series published by Boriani et al,^[2]^ the authors found no treatment failures in patients undergoing SAE, but incomplete treatment occurred frequently. In their study, 88% of spinal ABCs treated with SAE required at least 2 embolization procedures, with 35% of SAE treatments involving 6 or more embolization procedures. Therefore, we suggest that the classical application of SAE is as a preoperative therapy to decrease intraoperative bleeding and facilitate complete resection.

Although the role of radiotherapy in the treatment of ABC is controversial, considering the potential risks of growth restraint, post-radiation myelopathy, and malignant transformation, most authors do not suggest radiotherapy as the first treatment option for spinal ABC, especially in children.^[2,4,9]^ Radiotherapy is usually used as an adjunct to surgical treatment or in patients who cannot tolerate surgery.

Surgical resection is frequently considered the treatment of choice. The high rate of cure relates to the degree of excision.^[2,4,13]^ While en bloc resection with a negative margin may be more effective for overall local control, it is associated with greater morbidity and potential neurological sacrifice compared to intralesional curettage and thus may not be advisable in some cases.^[14]^ There are some reports about the postoperative complication after surgery, such as neurologic deterioration and massive bleeding. To prevent these complication, preoperative SAE is prerequisite and wide operative field exposure is also needed.

The recurrence of ABC, if it does recur, tends to happen early. Boriani et al^[12]^ reviewed 71 ABC cases and found that all recurrences happened within the first year after treatment. In general, skeletal ABCs have reported median recurrence rates ranging from 18 to 24 months.^[1,15,16]^ Because high rate of cure relates to the degree of excision, total excision of the lesion including entire cyst walls and spongy tissues is most important to obviate the risk of recurrence.

There are some special factors in adolescent patient with ABCs to be considered to decide treatment options. Adolescent have the highest incidence of postlaminectom kyphosis and the remaining growth of the spine. Therefore, instrumented fusion is inevitable. Radiotherapy is usually used as an adjunct to surgery. However, it has risk of radiation induced myelopathy, sarcoma and spinal deformity, especially in young patients.^[17–19]^

In this case, we performed anterior corpectomy and 360° instrumented fusion because the lesion showed circumferential involvement, and there was pathologic compression fracture of the thoracic vertebra. Although we performed preoperative SAE to prevent significant intraoperative bleeding, the tumor bled massively during anterior corpectomy, and the narrow operation field during the anterior approach made bleeding control difficult. However, during the posterior approach performed 1 week after the anterior approach, tumor bleeding was not severe, and it was easier to stop the bleeding than with the anterior approach. Due to the high probability of intraoperative bleeding, we performed a 2-stage operation.

## 4 Conclusion

Primary ABC of the spine is a benign lesion with a potential to be locally aggressive and a high rate of local recurrence. The optimal treatment of thoracic lesions is challenging due to their proximity to the spinal cord and nerve roots, and their frequent association with deformity. Surgical resection/curettage, SAE, and radiotherapy can be used alone or in combination. Complete exposure and resection is crucial to avoid the recurrence. Circumferential fusion and reconstruction of stability are also important for the treatment of thoracic ABC, especially in adolescent patient.

## Author contributions

**Conceptualization:** Jongpil Eun, Youngmin Oh.

**Data curation:** Jongpil Eun, Youngmin Oh.

**Formal analysis:** Youngmin Oh.

**Investigation:** Jongpil Eun, Youngmin Oh.

**Methodology:** Jongpil Eun, Youngmin Oh.

**Resources:** Youngmin Oh.

**Software:** Youngmin Oh.

**Supervision:** Youngmin Oh.

**Writing – original draft:** Jongpil Eun, Youngmin Oh.

**Writing – review & editing:** Jongpil Eun, Youngmin Oh.
